# A Collagen Membrane Pretreated with Citrate Promotes Collagen Mineralization and Bone Regeneration

**DOI:** 10.3390/jfb16070261

**Published:** 2025-07-15

**Authors:** Qi Zhang, Yewen Zhong, Xinlin He, Sui Mai

**Affiliations:** 1 Hospital of Stomatology, Sun Yat-sen University, Guangzhou 510055, China; 2 Guangdong Provincial Key Laboratory of Stomatology, Guangzhou 510080, China; 3 Institute of Stomatology, Sun Yat-sen University, Guangzhou 510080, China

**Keywords:** citrate, guided bone regeneration, carboxymethyl chitosan, intrafibrillar mineralization

## Abstract

**Purpose:** Collagen membranes with biomimetic mineralization are emerging as promising materials for bone regeneration, owing to their high biocompatibility. In this study, we developed a biogenic collagen membrane by combining citrate (C) pretreatment and carboxymethyl chitosan (CMC)-mediated mineralization and further evaluated its bone healing potential. **Methods:** C-CMC collagen membranes were prepared by lyophilization. The mineral composition and content were tested through X-ray diffraction (XRD), Fourier transform infrared (FTIR), and thermogravimetric analysis (TGA). The micromorphology was observed using transmission electron microscopy (TEM), scanning electron microscopy (SEM), and scanning probe microscopy (SPM). Physical and mechanical properties, including the swelling rate, porosity, hydrophilicity, tensile strength, Young’s modulus, degradation, and barrier function, were also evaluated. Bone mesenchymal stem cells (BMSCs) were cultured in vitro to observe their behavior. An in vivo critical-size rat calvarial defect model was used to validate the effects of the membrane on bone regeneration. **Results:** The C-CMC collagen membrane was successfully synthesized as a collagen–hydroxyapatite complex with intrafibrillar mineralization, exhibiting improved mechanical properties and an optimal swelling rate, porosity, hydrophilicity, and degradation rate. Additionally, the C-CMC collagen membrane promoted BMSC proliferation, adhesion, and osteogenesis while preventing epithelial cell infiltration. In vivo experiments indicated that C-CMC collagen membranes significantly stimulated bone regeneration without causing systemic toxicity. **Conclusions:** Our findings suggest that the C-CMC collagen membrane possesses satisfactory physical and mechanical properties, along with good biocompatibility and efficacy in bone defect regeneration, making it a potential candidate for a bioactive guided bone regeneration membrane in clinical applications.

## 1. Introduction

The regeneration of bone defects resulting from periodontitis, infection, or tumors remains a significant clinical challenge [1]. Guided bone regeneration (GBR) is a reliable technique for bone augmentation in the treatment of small bone defects [2]. Beyond the use of bone graft materials, GBR also requires the use of membrane materials to isolate bone defects from soft tissue [3], as well as providing space for bone regeneration [4,5].

Degradable GBR membranes are becoming increasingly popular, with tissue-derived collagen membranes being widely used in clinical practice because of the absence of secondary surgery requirements and their high biocompatibility [6,7]. Numerous studies have explored new strategies for incorporating bioactive compounds into membranes, with the aim of playing an active role in bone regeneration, rather than serving as a passive barrier. Bone regeneration is significantly influenced by the microenvironment at the defect site. As the GBR membrane constitutes an integral part of this microenvironment, collagen membranes that closely resemble natural bone can modulate stem cell differentiation, thereby contributing to bone regeneration to a certain degree.

To date, efforts have focused on combining collagen with minerals to refine scaffolds, thereby closely approximating the composition of the bone tissue. Nonetheless, accurately duplicating the microstructure of the native bone extracellular matrix (ECM), which entails the intra- and extrafibrillar mineralization of collagen fibers, poses a considerable challenge in advancing collagen-based materials. Our previous study confirmed that carboxymethyl chitosan (CMC) could successfully induce intrafibrillar mineralization, presented as hydroxyapatite (HA) deposited inside collagen fibers [8]. We attempted to prepare a biomimetic mineralized collagen scaffold based on this, but its degree of mineralization and pro-osteogenic properties still required further improvement. Accordingly, we propose a new strategy to pretreat the collagen membrane before mineralization to endow it with enhanced biogenic and physical properties via interfacial control between the organic matrix and inorganic minerals.

Citrate (C), a key component of biological hard tissue, such as bone, is believed to play an important role in both bone formation and osteoporosis therapy. It can reduce the interfacial energy between collagen fibers and amorphous calcium phosphate (ACP) in the early stages of mineralization and promote ACP precursor nucleation on collagen fibers through a wetting effect [9]. It can also bind closely to the hydroxyapatite surface and participate in the regulation of the mineral morphology and size [10]. Therefore, it has been widely used as a component of bone biomaterials to increase their mechanical and biological properties, but its role in biomineralization is not fully understood. Some studies suggest that a low concentration of citrate could induce the formation of HA on collagen membranes [11], but other studies have indicated that citrate may exert no influence on mineralization or potentially impede the nucleation and growth of HA by adsorbing to the mineral surface [12].

To determine the feasibility and biological effects of pretreatment with citrate, this study improved the biomimetic mineralization scheme by using a sodium citrate pretreatment collagen membrane before inducing collagen membrane biomimetic mineralization with CMC. We validated the physical, mechanical, and biocompatible properties, and the feasibility and safety of bone regeneration in calvarial defects, to provide an experimental basis for its long-term application in guiding bone regeneration technology.

## 2. Materials and Methods

### 2.1. Preparation of Collagen Membrane

The preparation of type I collagen was based on Price’s method [13]. Sprague-Dawley (SD) rat tail tendons were immersed in acetic acid (0.3 M) for three days to facilitate dissolution. Subsequently, the resulting collagen solution was centrifuged at 4 °C with a rotational speed of 3000 rpm for 30 min. The supernatant was then transferred to dialysis bags and dialyzed in a dipotassium phosphate solution (0.02 M) for 5–7 days. The collagen hydrogel obtained from the dialysis bags was rinsed with deionized water and lyophilized.

The lyophilized collagen was redissolved in 0.3 M acetic acid to a final concentration of 5 mg/mL. This solution was then mixed with phosphate-buffered saline (PBS), and the pH was adjusted to 7.2 using NaOH. The mixture was then poured into a mold at room temperature to obtain an even liquid film. Subsequently, the bottom of the mold was in direct contact with liquid nitrogen for 10 s while being open to air on top. The surface facing the bottom of the container was called the lower surface, and the opposite area was called the upper surface. Then, the membranes were frozen at −80 °C overnight and subsequently lyophilized. 

### 2.2. Preparation of CMC and C-CMC Collagen Membrane

**Figure 1:**
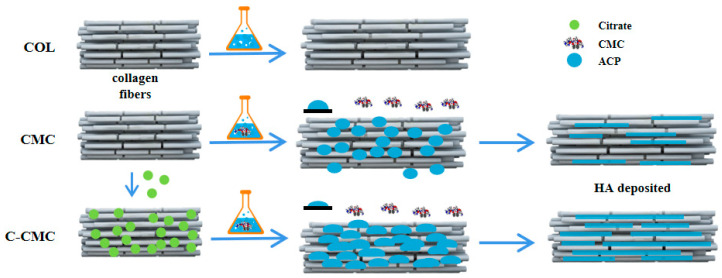
Schematic illustration of the remineralization procedure for collagen membranes via citrate pretreatment combined with CMC.

The C-CMC collagen membrane was prepared by immersing lyophilized collagen in a 5 M sodium citrate (Sigma-Aldrich, St. Louis, MO, USA) solution for 1 h, followed by rinsing in a biomimetic mineralization solution for 7 days. Both solutions were preadjusted to pH 7.4; the latter was a mixture of 4.5 mM CaCl2 and 2.1 mM K2HPO4, supplemented with 200 μg/mL of CMC (Mw: 150 kDa, RuibioC3105, Freiburg, Germany). The CMC collagen membrane was obtained by direct immersion in a biomimetic mineralization solution without sodium citrate pretreatment. The COL group served as a control and was rinsed with deionized water without any pretreatment (Figure 1). This study investigated the effects of the upper surface of a collagen membrane on osteogenesis.

### 2.3. Characterization of C-CMC Collagen Membrane

X-ray diffraction (XRD) patterns of the powder samples were acquired using an Empyrean X-ray diffractometer (Malvern Panalytical, Almelo, The Netherlands) over a 2θ range of 15–45° at a scanning speed of 2°/min. Fourier transform infrared spectroscopy (FTIR) with a Nicolet 6700 spectrometer (Thermo Fisher Scientific, Waltham, MA, USA) was employed to identify the functional groups, and the spectra were recorded at wavelengths of 400–4000 cm^−1^ with a resolution of 4 cm^−1^. Thermogravimetric analysis (TG) and derivative thermogravimetry (DTG), performed on a TG209F1 instrument (NETZSCH, Selb, Germany), were used to assess the thermal stability. The measurements were conducted at a heating rate of 5 °C per min in a nitrogen atmosphere, with temperatures ranging from ambient to 800 °C.

### 2.4. Electron Microscopy

The surface morphology of the collagen membrane was characterized by scanning electron microscopy (SEM, Regulus 8230, HITACHI, Tokyo, Japan) and scanning probe microscopy (SPM, Dimension Fastscan, Bruker, Berlin, Germany). The internal structure of the collagen membranes was analyzed by transmission electron microscopy (TEM, HT7800, HITACHI, Tokyo, Japan) at an accelerating voltage of 120 kV.

### 2.5. Mechanical Properties

The mechanical properties of the collagen membranes were assessed by tensile testing using a mechanical testing machine (Instron E3000, Norwood, MA, USA) at a stretching rate of 1 mm/min. The Young’s modulus was measured by the Si detector on the SPM instrument and then analyzed using NanoScope Analysis.

### 2.6. Swellling Rate, Porosity, and Degradation

Collagen membranes were immersed separately in saline for 5, 10, 30, 60, 90, and 120 min. The swelling rate was obtained using the following equation: swelling rate (%) = (W − W0)/W0 × 100, where W0 is the initial weight of the dry membrane, and W is the weight of the membrane after soaking.

The porosity of the collagen membranes was determined after soaking in absolute ethanol for 30 min using the following equation: porosity (%) = (M2 − M3 − M0)/(M1 − M3) × 100, where M0 is the initial weight of the collagen membrane, M1 is the mass of 5 mL of absolute ethanol and the centrifuge tube, M2 is the mass of the membrane soaked in absolute ethanol, and M3 is the weight of the tube and the remaining ethanol without the membrane.

The degradation experiment was performed using PBS, which was replaced every 24 h. Samples were collected and lyophilized on days 1, 7, 14, 21, 28, and 56, and the mass loss was calculated using the following equation: degradation percentage (%) = (M0 − M)/M × 100, where M0 is the initial weight of the membrane and M is the weight after degradation and lyophilization.

### 2.7. Water Contact Angle

Water contact angles were measured using a contact angle goniometer. A syringe was used to dispense water droplets onto the collagen membranes at a rate of 2.00 µL/s, and the static contact angles were documented. For each membrane, photographs were captured in three distinct areas and the resulting contact angles were averaged.

### 2.8. Barrier Function

Human gingival epithelial cells (HGECs) were generously provided by Professor Zhengmei Lin’s team at the Hospital of Stomatology, Sun Yat-sen University. Approximately 2 × 104 cells were seeded on the lower surface of the membrane, which was positioned at the base of the laser confocal culture dish (LCCDs, NEST, Wuxi, China), and cultured in DMEM supplemented with 10% FBS. The cells were stained with DAPI (C0065; Solarbio Science & Technology Co., Ltd., Beijing, China) on the 3rd day. Confocal laser scanning microscopy was used to capture images of both the upper and lower surfaces of the membrane.

### 2.9. Cell Culture

Rat bone marrow mesenchymal stem cells (BMSCs) were isolated from SD rats and cultured in DMEM/F-12 supplemented with 10% FBS) in a humidified atmosphere with 5% CO_2_ at 37 °C. The membranes were prewetted overnight in DMEM/F-12, and BMSCs were seeded onto the membranes at a certain concentration.

### 2.10. Cell Proliferation and Adhesion

Cell proliferation was assessed by the co-culture of BMSCs with collagen membranes. At predetermined times (1, 3, 5, and 7 days), 10% Cell Counting Kit 8 solution (CCK-8, Dojindo, Kumamoto, Japan) was added to each well and incubated in the cell incubator for another one hour, and the medium was then transferred to 96 well-plates. The optical density (OD) of each well was measured at a wavelength of 450 nm.

To observe the cell morphology, cells in the LCCDs were fixed with 4% paraformaldehyde for 10 min following a 2-day cultivation period, after which they were permeabilized using a 0.5% Triton X-100 solution (Invitrogen, Carlsbad, CA, USA). The primary antibody, rabbit monoclonal anti-vinculin (1:800 dilution, catalog number 8814; CST, Boston), was applied and incubated for 14 h at 4 °C. Subsequently, Alexa Fluor 488-conjugated goat anti-rabbit IgG secondary antibodies (1:200 dilution; catalog number S0018; Affinity, Changzhou, China) were added and incubated for 1 h. The samples were labeled with FITC–phalloidin (catalog number CA1610; Solarbio Science & Technology Co., Ltd., Beijing, China) and DAPI (catalog number C0065; Solarbio Science & Technology Co., Ltd., Beijing, China). Imaging was performed using a FV3000 laser scanning confocal microscope (LSCM, Olympus, Tokyo, Japan).

### 2.11. ALP Staining

Osteogenic induction medium was supplemented with 0.1 μM dexamethasone, (50 μg/mL) and β-sodium glycerophosphate (10 mM), all of which were obtained from Sigma-Aldrich (St. Louis, MO, USA). Following a 14-day incubation period, the cells were fixed for 30 min and subsequently stained for 30 min according to the protocol provided by the ALP staining kit (PH Biotechnology, Shanghai, China). The cell morphology was visualized using an optical microscope.

### 2.12. Calvarial Defected Model Construction

A total of 24 male SD rats, aged 8–10 weeks and weighing approximately 250 g, were procured from the Sun Yat-sen University Laboratory Animal Center in China. The Institutional Animal Care and Use Committee of Sun Yat-sen University approved all animal experiments, which were conducted in strict accordance with the approved protocol (No. SYSU-IACUC-2022-002623). Animals were randomly divided into four groups: COL, CMC, C-CMC, or CTRL group (*n* = 6). Surgical procedures were performed under general anesthesia induced by 1% pentobarbital sodium at a dose of 50 mg/kg. A longitudinal incision was made in the middle of the skull, and the skin was retracted laterally to scrape off the underlying fascia and expose the calvarium. Subsequently, a dental trephine (d = 5 mm) was used to create a 5 mm circular full-thickness skull defect, supplemented with sterile saline irrigation to maintain the temperature [14]. The defect was covered with the upper surface of the COL, CMC, or C-CMC collagen membrane or left empty (CTRL), and the surgical incision was closed with a 3-0 suture. After the rats had awoken and returned to normal conditions, the feeding conditions and incision healing were observed. The rats were euthanized 4 and 8 weeks post-surgery (*n* = 3 at each time points) [15]. The skulls and organs were harvested in paraformaldehyde for 48 h and transferred to 70% ethanol for micro-CT and histological examination.

### 2.13. Systemic Toxicity Assessment

The organ samples, including the liver, spleen, and kidney, were dehydrated using an alcohol gradient, embedded in paraffin, and sectioned into 4-μm-thick slices. These sections were stained with hematoxylin and eosin (HE) and subsequently observed and photographed under an optical microscope.

### 2.14. Micro-Computed Tomography (Micro-CT)

The skulls were trimmed to a size of approximately 2 cm × 2 cm, containing the defect area, and subsequently scanned using micro-CT (Micro-CT 50, SCANCO Medical, Zurich, Switzerland) at a voltage of 70 kV and a current of 200 µA. The region of interest (ROI) was defined as a circular area with a diameter of 5 mm where the defect was located. The 3D reconstructions of the samples were obtained using the RadiAnt DICOM Viewer software(version 2021.2.2). The bone volume/total volume ratio (BV/TV) and the trabecular thickness (Tb.Th) were also quantitatively analyzed.

### 2.15. Histological Assay

After scanning, the samples were immersed in ethylenediaminetetraacetic acid solution for 14 days. After decalcification, gradient dehydration was performed using alcohol and paraffin-embedded sections. HE staining was performed according to the manufacturer’s instructions. Osteocalcin (OCN) was detected by immunohistochemical staining. Images were captured using a slice scanner (Aperio AT2; Leica Biosystems, Vista, CA, USA). The ImageJ software (version 1.51k; National Institutes of Health) was used to quantify the optical density from three randomly selected images per group.

### 2.16. Statistical Analysis

Data are presented as the mean  ±  standard deviation, derived from at least three independent experiments. Statistical analyses were conducted using GraphPad Prism (version 9) with one-way analysis of variance (ANOVA), followed by Bonferroni’s post hoc test for multiple comparisons, assuming homogeneity of variance. *p*  <  0.05 was considered to indicate statistical significance.

## 3. Results

### 3.1. Characterization of C-CMC Collagen Membrane

**Figure 2:**
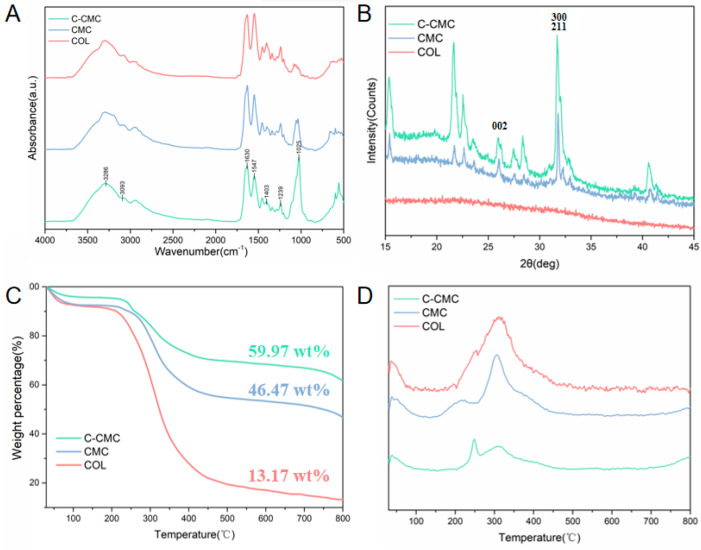
Mineral composition and content of COL, CMC, and C-CMC membranes. (**A**) FTIR spectra. (**B**) XRD spectra. (**C**) TGA patterns. (**D**) DTG patterns.

The FTIR results revealed distinct absorption peaks characteristic of collagen amide bands in the collagen membranes within the following ranges: 3310–3270 cm^−1^ (amide A), 3100–3030 cm^−1^ (amide B), 1650–1630 cm^−1^ (amide I), 1550–1535 cm^−1^ (amide II), and approximately 1240 cm^−1^ (amide III). An increase in phosphate (~1020 cm^−1^) and carbonate (~1410 cm^−1^) functionalities was identified in the CMC and C-CMC groups, associated with carbonated calcium phosphate (CaP), as shown in Figure 2A. The CaP phase was confirmed to be poorly crystalline apatite by X-ray diffraction (XRD), as depicted in Figure 2B. The temperature change curve (Figure 2C) showed that the mass of each group decreased with increasing temperatures. The final mass of the C-CMC collagen membrane was 59.97%, which was higher than that of the CMC (46.47%) and COL (13.17%) groups. An analysis of the DTG curve (Figure 2D) revealed three desorption peaks for the C-CMC group. The weightlessness peak at 30–100 °C may be related to the decomposition of water molecules inside the collagen fiber, the weightlessness peak at 200–400 °C may be related to the decomposition of the collagen organic matrix, and the weightlessness peak at 700–800 °C may be related to the release of CO_2_ at high temperatures of carbonate apatite [16]. These results validate the presence of more minerals in the C-CMC collagen membrane.

### 3.2. Micromorphology of C-CMC Collagen Membrane

**Figure 3:**
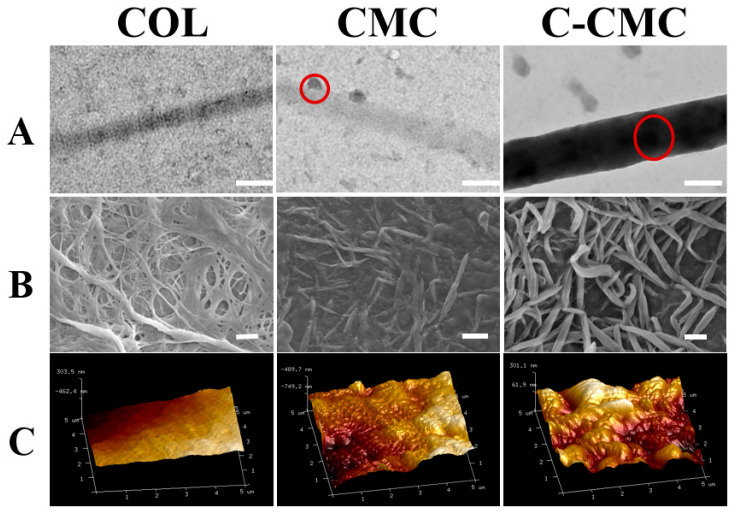
The morphology of collagen fibers and the membrane surface. (**A**) TEM images of collagen fibers; the red circle indicates the crystal deposits. Scale bar: 1 μm. (**B**) SEM image showing the surface of the collagen membrane. Scale bar: 10 μm. (**C**) SPM images of different membrane surfaces.

The morphological structure of the collagen fibers was observed using TEM (Figure 3A). A typical transverse pattern structure was observed in the COL collagen fibers but not in the CMC group. Moreover, the electron density increased and apatite occurred on the fibers in the CMC group. The electron density was more uniform and higher in C-CMC collagen, indicating intrafibrillar mineralization. SEM (Figure 3B) and SPM (Figure 3C) were used to observe the micromorphology of the collagen membrane surface, which showed that the collagen membrane surface of the COL group was smooth with a loose pore structure. SEM showed that the pores of the CMC group were filled with apatite, which was consistent with the irregular protrusions observed in SPM. The fibers were larger in the C-CMC group, and the pore structure was more obvious in SEM, whereas SPM showed a rougher surface with swollen collagen.

### 3.3. Physical and Mechanical Properties of C-CMC Collagen Membrane

**Figure 4:**
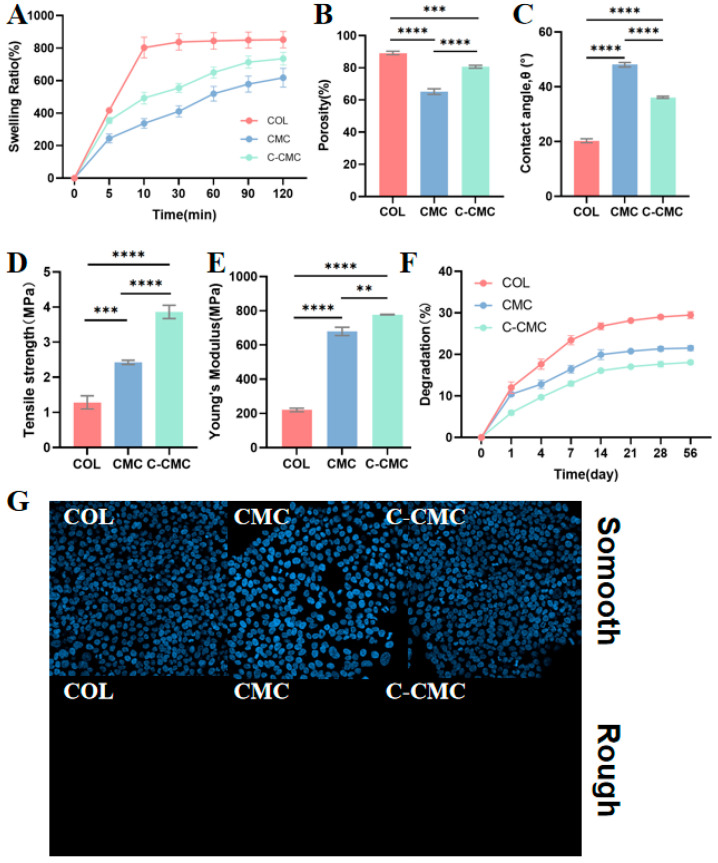
Physical and mechanical properties of pure, CMC, and C-CMC collagen membranes. (**A**) Swelling rate. (**B**) Porosity. (**C**) Water contact angle. (**D**) Tensile strength. (**E**) Young’s modulus. (**F**) Degradation in PBS. (**G**) Representative fluorescent images of HGECs, which were labeled with DAPI, cultured on the membrane surface, and images of the opposite side (** *p* < 0.005; *** *p* < 0.001; **** *p* < 0.0001).

The swelling rate (Figure 4A) of the COL group (657.8%) was the highest among all groups, whereas the swelling rate of C-CMC (500.3%) was higher than that of CMC (387.1%), with no statistically significant difference. The porosity (Figure 4B) of the COL group (89.14%) was also the highest, and that of the CMC group (65.23%) was significantly lower than that of the C-CMC group (80.66%) (*p* < 0.005). The water contact angle (Figure 4C) of the COL group was the lowest (20.28°), and mineralization with CMC significantly decreased the hydrophilicity (48.07°). The use of citrate restored the hydrophilicity to some extent (36.15°). The tensile strength (Figure 4D) of C-CMC (3.862 N) was significantly higher than that of CMC (2.425 N) and COL (1.285 N) (*p* < 0.001). The Young’s modulus (Figure 4E) of C-CMC (778 GPa) was also significantly improved compared with those of CMC (679.7 GPa) and COL (220.2 GPa) (*p* < 0.005). The degradation rates (Figure 4F) of CMC and COL showed no significant difference on the first day. In the COL group, the quality consistently decreased to 20.8% within 56 days, whereas that of the CMC and C-CMC groups decreased after 7 days, and the final degradation rates were approximately 15.4% and 12.17%, respectively, which were statistically significantly different (*p* < 0.005). The physical barrier function of the HGECs was tested in vitro. Figure 4G shows that no cells penetrated the opposite side of the collagen membrane after 72 h.

### 3.4. C-CMC Collagen Membrane Promotes Adhesion and Osteogenic Differentiation of BMSCs In Vitro

**Figure 5:**
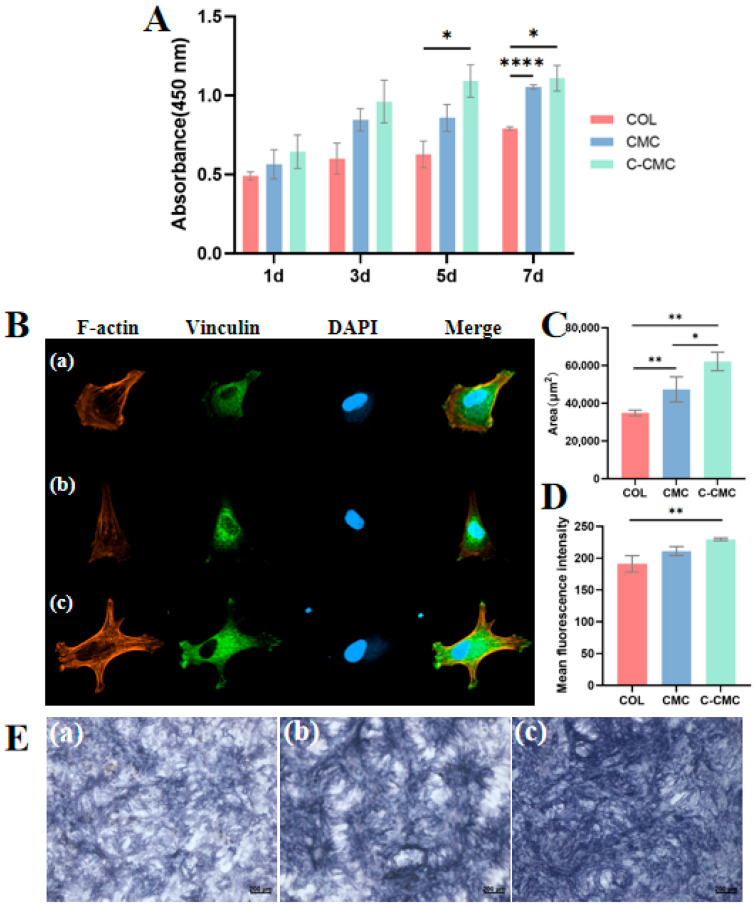
Proliferation, adhesion, and osteogenesis of BMSCs on COL, CMC, and C-CMC membranes. (**A**) Cell proliferation evaluated by CCK8 assay. (**B**) Adhesion and morphology of BMSCs on different collagen membranes. (F-actin: red; vinculin: green; DAPI: blue) Magnification: 400×. (**C**) Cell areas and (**D**) mean fluorescence intensity of vinculin for BMSCs seeded on different collagen membranes. (**E**) ALP staining of BMSCs co-cultured with collagen membranes for 14 days. (**a**–**c**) represent the COL, CMC, and C-CMC groups, respectively (* *p* < 0.05, ** *p* < 0.01, **** *p* < 0.0001).

No cytotoxicity was observed in any group (Figure 5A). In particular, the viability of the C-CMC group was significantly lower than that of the COL group on the 5th day. In addition, the CMC and C-CMC groups exhibited better cell proliferation abilities on the 7th day.

Semi-quantitative analysis revealed significant differences in the BMSCs’ morphology and adhesion across membrane types (Figure 5B–D). From the F-actin channel, which marked the cytoskeleton, cells cultured on C-CMC membranes exhibited the most extensive spreading, with a polygonal osteoblast-like morphology featuring more pseudopodia and an average cell area of 62,124 ± 4886 μm^2^. This represented an increase in the spreading area compared to CMC membranes (47,395 ± 6669 μm^2^; *p* < 0.01) and COL membranes (34,895 ± 1482 μm^2^; *p* < 0.05). In addition, the fluorescence intensity of vinculin, a marked adhesion protein, was detected in all three scaffold groups, with the strongest fluorescence intensity observed in the C-CMC group, although with no significant difference compared to the CMC group. The above results indicate that the C-CMC collagen membrane may be conducive to the maximum spreading of BMSCs and promotes cell adhesion.

The osteogenic differentiation of BMSCs on the collagen membrane was visualized using ALP staining (Figure 5E). The results showed that both the CMC and C-CMC groups were stained, but the extent in C-CMC was higher than that in the CMC group.

### 3.5. C-CMC Collagen Membrane Promotes In Situ Bone Regeneration

**Figure 6:**
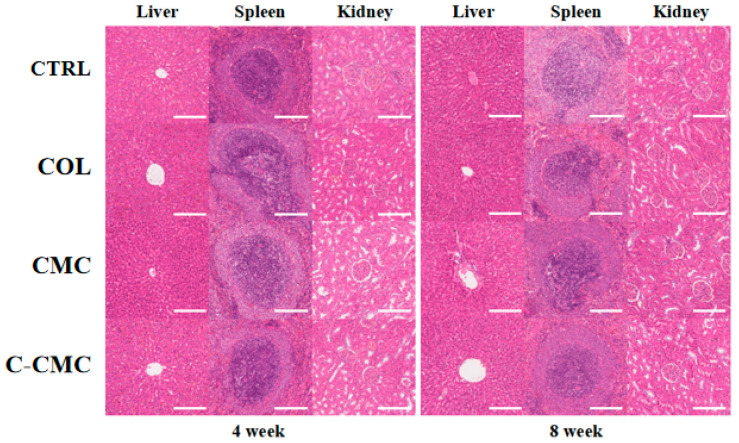
Systematic toxicity of blank control, COL, CMC, and C-CMC collagen membranes after 4 or 8 weeks. Magnification: 20×. Scale bar: 500 μm.

The microscopic examination of all specimens revealed no toxic reactions in the liver, spleen, or kidney tissue of SD rats at 4 and 8 weeks post-implantation (Figure 6).

**Figure 7:**
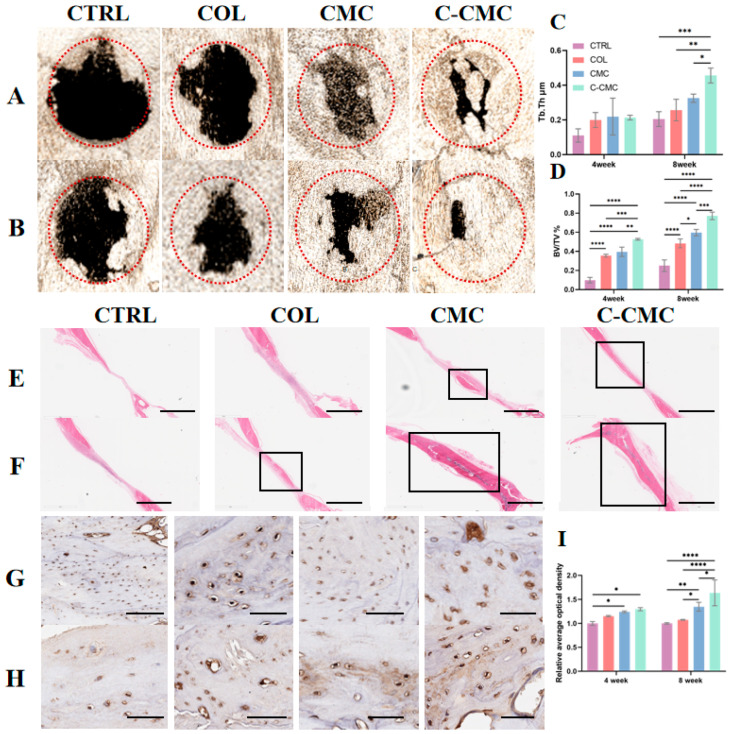
In vivo bone regeneration in calvarial defects. (**A**,**B**) The 3D reconstructions of rat craniums in 4 groups after being implanted for (**A**) 4 weeks and (**B**) 8 weeks. Dash circles indicate the region of interest. (**C**) The mean trabecular thickness (Tb.Th). (**D**) The bone volume fraction (BV/TV). (**E**,**F**) HE staining of rat calvarial defects at (**E**) 4 weeks and (**F**) 8 weeks. Black boxes indicate new bone formation. Scale bar: 300 μm. (**G**,**H**) OCN staining of calvarial defects at (**G**) 4 weeks and (**H**) 8 weeks. Scale bar: 50 μm. (**I**) The relative average optical density of OCN staining (* *p* < 0.05, ** *p* < 0.01, *** *p* < 0.001, **** *p* < 0.0001).

Micro-CT scans of calvarial bone defects, which were either left untreated (CTRL) or covered with COL, CMC, or C-CMC collagen membranes, are presented for 4 and 8 weeks (Figure 7A,B). Coronal sections at 4 weeks showed that new bone tissue could be seen in the center of the bone defects in the C-CMC group, whereas only some new bone formation was observed at the margins of the bone defect area in the CTRL and COL groups, which was significantly smaller than that in the CMC group. Eight weeks after implantation, new bone formation increased in all four groups, and the bone defect was almost complete in the C-CMC group, where it was more obvious than that in the CTRL and COL groups. An analysis of the 3D reconstruction indicated that the BV/TV ratio (Figure 7D) was significantly higher in the C-CMC group than in the other groups after 4 or 8 weeks (both *p* < 0.05), whereas Tb.Th (Figure 7C) showed significant differences until 8 weeks.

Histological analysis, as depicted in Figure 7E–H, confirmed the bone formation observed in the micro-CT scans. At 4 weeks, the bone defect implanted with a COL membrane or left untreated was filled with a thin layer of connective fibrous tissue, with no apparent signs of bone formation. In contrast, new bone formation was evident in defects implanted with CMC and C-CMC membranes, and better continuity of the newly formed bone was observed in the C-CMC group at four weeks (Figure 7E). At 8 weeks, new bone formation was observed in the COL group, whereas the defect was almost complete in the CMC and C-CMC groups (Figure 7F). The OCN staining results (Figure 7G–H) and the semi-quantitative analysis of the relative optical density (Figure 7I) were consistent with the HE staining. At 4 weeks, both the CMC and C-CMC groups showed significantly higher OCN expression than CTRL (*p* < 0.05), although no statistically significant difference existed between these two groups (*p* > 0.05). By 8 weeks, the OCN levels in the CMC and C-CMC groups had further increased, significantly exceeding both the CTRL and COL groups (*p* > 0.05). Notably, C-CMC membranes induced higher OCN expression than CMC (*p* < 0.05), correlating with mature bone matrix deposition. These results indicate the stimulatory potential of C-CMC membranes for in situ bone regeneration.

## 4. Discussion

In guided bone regeneration, barrier membranes are essential for preventing soft tissue invasion and promoting bone regeneration. Consequently, these membranes must possess sufficient mechanical strength and appropriate bioactivity to satisfy clinical requirements [16]. In biomineralized tissue, apatite microcrystals are deposited in an orderly fashion within collagen fibers. This not only enhances the mechanical properties of the collagen matrix but also creates an osteoinductive microenvironment, which is crucial for the osteogenic differentiation of mesenchymal stem cells [17]. To better mimic the biomineral formation of HA in the bone tissue, citrate was used to pretreat the collagen membrane before CMC-mediated mineralization to prevent the potential inhibitory effect of free citrate added directly to the mineralized solution on HA mineral nucleation and growth.

The characteristic absorption peaks of collagen and HA were found in both the CMC and C-CMC groups, indicating that the biomimetic mineralized collagen membranes were collagen–HA composites. Moreover, the characteristic HA diffraction peak in the XRD pattern confirmed the formation of an HA crystal mineral phase in the mineralized collagen membranes. The TGA results suggest a positive effect of citrate pretreatment on the mineralization effect.

The effect of citrate pretreatment on mineralization was further verified by electron microscopy. TEM images showed that the electron density of collagen fibers in the C-CMC group was significantly increased, the collagen fibers were thickened, and no mineral deposition was observed on the surface. This suggested the occurrence of intrafibrillar mineralization in the C-CMC collagen membrane because it had obviously different images compared to the CMC group, which showed crystals deposited outside the fibers. Similarly, SEM images showed that the fibers were thicker in the C-CMC collagen membrane than in the CMC. The SPM results were similar to those of the SEM, with the appearance of more prominent surface protrusions. The distinction between citrate-pretreated (C-CMC) and non-pretreated (CMC) mineralization lies in their mineralization patterns and resulting structural hierarchies. Citrate pretreatment critically redirects mineral deposition from extrafibrillar to intrafibrillar sites, while CMC cannot fully induce intrafibrillar mineralization, resulting in superficial apatite aggregates on collagen fibers (Figure 3B) and pore occlusion.

Our characterization revealed that citrate pretreatment restructured the collagen–mineral interfaces, and the citrate-mediated intrafibrillar mineralization in C-CMC membranes conferred functionally distinct advantages. First, the structural integrity was enhanced through homogeneous hydroxyapatite incorporation within collagen fibers. This intrafibrillar mineralization pattern increased the fiber diameter compared to pristine collagen (COL) while preserving the interconnected porosity, with a value within the optimal range for nutrient diffusion and cell infiltration [18]. Second, the mechanical properties were optimized via mineral–collagen synergy, yielding significantly higher tensile strength and Young’s moduli while maintaining balanced hydrophilicity. The C-CMC membrane exhibited an appropriate swelling rate for clinical use, avoiding the obstructions to surgical implantation and long-term in vivo retention [19] caused by COL membranes with excessively high swelling rates. Third, the biodegradation kinetics were modulated by denser mineralization, slowing mass loss of 12.2% versus 15.4% in CMC at 56 days. Furthermore, the increased mechanical strength and appropriately extended degradation rate indicated better spatial maintenance and barrier function in vivo, consistent with the results of the barrier function assay. Critically, citrate-mediated intrafibrillar mineralization reconciles traditionally competing requirements, i.e., offering a high mineralization degree without sacrificing porosity or flexibility.

The ability to recruit stem cells to bone defect areas is a prerequisite for better bone regeneration [20]. Biocompatibility was confirmed using the CCK-8 assay. CLSM staining suggested that the C-CMC collagen membrane significantly promoted cell adhesion. For example, the highest focal spot protein fluorescence intensity and largest cell area were observed in the C-CMC group. This superior adhesion profile might stem from the citrate-mediated optimization of the material’s physicochemical properties, which collectively mimic the osteogenic microenvironment of native bone. Cell adhesion is often affected by the chemical composition and morphology [21]. Three interconnected features might contribute to this behavior. First, the morphological complexity of C-CMC membranes provided critical anchor points for filopodia extension, directly facilitating pseudopodia formation and cytoskeletal engagement. Second, enhanced hydrophilicity, attributable to citrate-derived carboxyl groups, promoted the adsorption of adhesion proteins like fibronectin [22]. Third, the optimized stiffness approached the mechanical range of trabecular bone, activating mechanotransduction pathways that drive vinculin clustering. The C-CMC membranes established a biomimetic interface that integrated chemical, topographical, and mechanical cues, effectively bridging extracellular signaling to intracellular responses [23,24]. This might explain their exceptional capacity to support osteoblast-like spreading and adhesion complex assembly, accelerating subsequent bone regeneration processes.

Our integrated findings demonstrated that citrate pretreatment conferred both structural and bioactive superiority to the C-CMC membrane, enabling the stage-specific enhancement of bone regeneration. Early in vitro ALP staining revealed significantly elevated osteogenic differentiation in BMSCs cultured on C-CMC membranes compared to both the CMC and COL groups, indicating citrate’s role in initiating osteoblast maturation. This early pro-osteogenic effect translated to in vivo outcomes: micro-CT and HE staining confirmed the near-complete defect healing in the C-CMC group by 8 weeks, while the control groups exhibited limited bone formation confined to defect margins. This suggests that the C-CMC collagen membrane is not only a passive barrier but can also play an active role in bone regeneration [24]. The temporal progression of osteogenesis was further elucidated by the OCN dynamics. While the CMC and C-CMC groups showed comparable OCN expression at 4 weeks, C-CMC induced higher OCN levels than CMC by 8 weeks (*p* < 0.05), correlating with mature bone matrix deposition [25].

This functional divergence underscores citrate’s dual mechanism of action in bone regeneration. First, citrate-mediated intrafibrillar mineralization established a bone-mimetic microenvironment that perpetuated mechanical stimulation for osteoblast differentiation, thereby accounting for the early ALP elevation observed in our study [26]. Concurrently, the gradual release of citrate during membrane degradation activated the SLC13A5 transporter, which upregulated ALP expression, as demonstrated in vitro, while simultaneously potentiating late-stage OCN synthesis through enhanced cellular energy metabolism. This synergistic mechanism distinguished C-CMC from passive barriers [27].

It is becoming increasingly evident that metabolism plays a crucial role as a regulator, interacting with various signaling pathways and epigenetic networks to modulate cell proliferation, differentiation, and physiological responses [28]. Citrate, a well-characterized intermediate metabolite, plays a pivotal role in regulating energy homeostasis [28,29]. Recent studies have identified a correlation between citrate metabolism and bone formation. The plasma membrane transporter SLC13A5, which is responsible for citrate transport, exhibits significantly increased gene expression in newly formed rat bone and during the early stages of osseointegration [30]. SLC13A5 deficiency is associated with impaired bone formation and enamel development anomalies [31]. However, the regulatory role of citrate as a degradation product in cellular functions has not yet been fully explored. It has recently been shown that citrate, either loaded with biomaterials or added as an exogenous supplement, can promote bone development by upregulating alkaline phosphatase genes and accelerating the phenotypic progression of osteoblasts. Therefore, it is of interest to determine whether the C-CMC collagen membrane can promote bone formation via citrate release during the degradation of metabolic pathways.

## 5. Conclusions

This study established citrate pretreatment as a strategic approach to engineer collagen membranes with biomimetic intrafibrillar mineralization. The fabricated C-CMC collagen membranes possessed optimized physical and mechanical properties, rendering them more suitable for clinical applications. In particular, the C-CMC collagen membrane exhibited superior biocompatibility and pronounced enhancements in bone regeneration without systemic toxicity, both in vitro and in vivo. Our study demonstrates that C-CMC membranes transcend passive barrier functions by actively orchestrating bone regeneration. Citrate-mediated intrafibrillar mineralization thus represents a promising strategy for next-generation bioactive membranes.

## Data Availability

The original contributions presented in this study are included in the article; further inquiries can be directed to the corresponding author.

## References

[1] Li N., Wang J., Feng G., Liu Y., Shi Y., Wang Y., Chen L. (2024). Advances in biomaterials for oral-maxillofacial bone regeneration: Spotlight on periodontal and alveolar bone strategies. Regen. Biomater..

[2] Ali M., Mohd Noor S.N.F., Mohamad H., Ullah F., Javed F., Abdul Hamid Z.A. (2024). Advances in guided bone regeneration membranes: A comprehensive review of materials and techniques. Biomed. Phys. Eng. Express.

[3] Qiu W., Zhang K., Wu M., Fu M., Wu H., Tang R., Chen Z., Guo J., Fang F. (2025). Tri-Layer Citrate-Based Hydroxyapatite Composite Scaffold Promoting Osteogenesis and Gingival Tissue Regeneration for Periodontal Bone Defect Repair. Adv. Healthc. Mater..

[4] Yan F., Yu M., He Y., Wang F., Yang F., Zhao X., Zheng Y., Liu Y., Xia D., Liu Y. (2024). Hierarchical Mineralized Collagen Coated Zn Membrane to Tailor Cell Microenvironment for Guided Bone Regeneration. Adv. Funct. Mater..

[5] Yin L.H., Wang K.J., Lv X.Q., Sun R.Q., Yang S.H., Yang Y.J., Liu Y.Y., Liu J.T., Zhou J., Yu Z.H. (2017). The fabrication of an ICA-SF/PLCL nanofibrous membrane by coaxial electrospinning and its effect on bone regeneration in vitro and in vivo. Sci. Rep..

[6] Ren Y., Fan L., Alkildani S., Liu L., Emmert S., Najman S., Rimashevskiy D., Schnettler R., Jung O., Xiong X. (2022). Barrier Membranes for Guided Bone Regeneration (GBR): A Focus on Recent Advances in Collagen Membranes. Int. J. Mol. Sci..

[7] Sheikh Z., Qureshi J., Alshahrani A.M., Nassar H., Ikeda Y., Glogauer M., Ganss B. (2017). Collagen based barrier membranes for periodontal guided bone regeneration applications. Odontology.

[8] Wang R.X., Guo J.X., Lin X.X., Chen S.P., Mai S. (2020). Influence of molecular weight and concentration of carboxymethyl chitosan on biomimetic mineralization of collagen. RSC Adv..

[9] Shao C.Y., Zhao R.B., Jiang S.Q., Yao S.S., Wu Z.F., Jin B., Yang Y.L., Pan H.H., Tang R.K. (2018). Citrate Improves Collagen Mineralization via Interface Wetting: A Physicochemical Understanding of Biomineralization Control. Adv. Mater..

[10] Ruiz-Agudo E., Ruiz-Agudo C., Di Lorenzo F., Alvarez-Lloret P., Ibañez-Velasco A., Rodriguez-Navarro C. (2021). Citrate Stabilizes Hydroxylapatite Precursors: Implications for Bone Mineralization. ACS Biomater. Sci. Eng..

[11] Rhee S.H., Tanaka J. (1999). Effect of citric acid on the nucleation of hydroxyapatite in a simulated body fluid. Biomaterials.

[12] Wu X., Dai H., Yu S., Zhao Y., Long Y., Li W., Tu J. (2021). Citrate regulates extracellular matrix mineralization during osteoblast differentiation in vitro. J. Inorg. Biochem..

[13] Rajan N., Habermehl J., Coté M.F., Doillon C.J., Mantovani D. (2006). Preparation of ready-to-use, storable and reconstituted type I collagen from rat tail tendon for tissue engineering applications. Nat Protoc..

[14] Kim W.J., Ryu J.H., Kim J.W., Kim K.T., Shin H.R., Yoon H., Ryoo H.M., Cho Y.D. (2024). Bone-targeted lipoplex-loaded three-dimensional bioprinting bilayer scaffold enhanced bone regeneration. Regen. Biomater..

[15] Liu X., Chen W., Shao B., Zhang X., Wang Y., Zhang S., Wu W. (2021). Mussel patterned with 4D biodegrading elastomer durably recruits regenerative macrophages to promote regeneration of craniofacial bone. Biomaterials.

[16] Wu Y., Chen S.C., Luo P., Deng S.D., Shan Z.J., Fang J.H., Liu X.C., Xie J.X., Liu R.H., Wu S.Y. (2022). Optimizing the bio-degradability and biocompatibility of a biogenic collagen membrane through cross-linking and zinc-doped hydroxyapatite. Acta Biomater..

[17] Sauro S., Babbar A., Gharibi B., Feitosa V.P., Carvalho R.M., Rodrigues L.K.A., Banerjee A., Watson T. (2018). Cellular differentiation, bioactive and mechanical properties of experimental light-curing pulp protection materials. Dent. Mater..

[18] Hollister S.J. (2005). Porous scaffold design for tissue engineering. Nat. Mater..

[19] Wang X., Li X., Zhang Z. (2010). Investigation on preparation and property of collagen-hydroxyapatite composite membrane for guided bone regeneration. Chem. Res. Appl..

[20] Armiento A.R., Hatt L.P., Rosenberg G.S., Thompson K., Stoddart M.J. (2020). Functional Biomaterials for Bone Regeneration: A Lesson in Complex Biology. Adv. Funct. Mater..

[21] Xiao L., Sun Y., Liao L., Su X. (2023). Response of mesenchymal stem cells to surface topography of scaffolds and the underlying mechanisms. J. Mater. Chem. B.

[22] Toffoli A., Parisi L., Bianchi M.G., Lumetti S., Bussolati O., Macaluso G.M. (2020). Thermal treatment to increase titanium wettability induces selective proteins adsorption from blood serum thus affecting osteoblasts adhesion. Mater. Sci. Eng. C.

[23] Morelli S., D′Amora U., Piscioneri A., Oliviero M., Scialla S., Coppola A., De Pascale D., Crocetta F., De Santo M.P., Davoli M. (2024). Methacrylated chitosan/jellyfish collagen membranes as cell instructive platforms for liver tissue engineering. Int. J. Biol. Macromol..

[24] Yang D., Xu Z., Huang D., Luo Q., Zhang C., Guo J., Tan L., Ge L., Mu C., Li D. (2025). Immunomodulatory multifunctional janus collagen-based membrane for advanced bone regeneration. Nat. Commun..

[25] Li J., Zhang H., Yang C., Li Y., Dai Z. (2016). An overview of osteocalcin progress. J. Bone Miner. Metab..

[26] Liu P., Tu J., Wang W., Li Z., Li Y., Yu X., Zhang Z. (2022). Effects of Mechanical Stress Stimulation on Function and Expression Mechanism of Osteoblasts. Front. Bioeng. Biotechnol..

[27] Xu H., Yan S., Gerhard E., Xie D., Liu X., Zhang B., Shi D., Ameer G.A., Yang J. (2024). Citric Acid: A Nexus Between Cellular Mechanisms and Biomaterial Innovations. Adv. Mater..

[28] Baker S.A., Rutter J. (2023). Metabolites as signalling molecules. Nat. Rev. Mol. Cell Biol..

[29] Liu Q., Xue Y., Guo J., Tao L., Zhu Y. (2025). Citrate: A key signalling molecule and therapeutic target for bone remodeling disorder. Front. Endocrinol..

[30] Roosa S.M.M., Liu Y.L., Turner C.H. (2011). Gene Expression Patterns in Bone Following Mechanical Loading. J. Bone Min. Res..

[31] Irizarry A.R., Yan G.R., Zeng Q.Q., Lucchesi J., Hamang M.J., Ma Y.F.L., Rong J.X.J. (2017). Defective enamel and bone development in sodium-dependent citrate transporter (NaCT) deficient mice. PLoS ONE.

